# Posture Control of Hydraulic Flexible Second-Order Manipulators Based on Adaptive Integral Terminal Variable-Structure Predictive Method

**DOI:** 10.3390/s25051351

**Published:** 2025-02-22

**Authors:** Jianliang Xu, Zhen Sui, Feng Xu

**Affiliations:** 1 School of Mechanical and Electrical Engineering, Quzhou College of Technology, Quzhou 324000, China; xujianliang84@gmail.com (J.X.); fengxu15@mails.jlu.edu.cn (F.X.); 2 College of Communication Engineering, Jilin University, Changchun 130022, China

**Keywords:** manipulator, sliding mode control, predictive control, posture trajectory tracking, disturbance compensation, multi-input multi-output system

## Abstract

As operational scenarios become more complex and task demands intensify, the requirements for the intelligence and automation of manipulators in industry are increasing. This work investigates the challenge of posture tracking control for hydraulic flexible manipulators by proposing a discrete-time integral terminal sliding mode predictive control (DITSMPC) method. First, the proposed method develops a second-order dynamic model of the manipulator using the Lagrangian dynamic strategy. Second, a discrete-time sliding mode control (SMC) law based on an adaptive switching term is designed to achieve high-precision tracking control of the system. Finally, to weaken the influence of SMC buffeting on the manipulator system, the predictive time domain function is integrated into the proposed SMC law, and the delay estimation of the unknown term in the manipulator system is carried out. The DITSMPC scheme is derived and its convergence is proven. Simulation experiments comparing the DITSMPC scheme with the classical discrete-time SMC method demonstrate that the proposed scheme results in smooth torque changes in each joint of the manipulator, with the integral of torque variations being $5.22\times 10^{3}$. The trajectory tracking errors for each joint remain within ±0.0025 rad, all of which are smaller than those of the classical scheme.

## 1. Introduction

An industrial manipulator is a mechanical device designed to emulate the movement of the human arm and is extensively applied in industries such as healthcare, manufacturing, transportation, and services. It typically comprises multiple joints and connecting rods, with movement controlled by electric motors, pneumatic systems, or hydraulic systems. The design and functionality of manipulators can be customized for specific applications, offering varying degrees of freedom and operational ranges [1]. Compared with traditional manual operations, industrial manipulators provide unparalleled advantages in complex and hazardous working environments, particularly those involving high temperatures, high pressures, or dangerous materials. Their precision, efficiency, and adaptability make them indispensable in such scenarios [2]. However, despite these advantages, real-world applications of robotic arms face significant challenges. Factors such as external environmental disturbances, load variations, and internal disturbances often cause fluctuations in system model parameters, which negatively impact the accuracy and stability of a robotic arm’s movement trajectory [3,4]. This problem becomes especially pronounced in unstructured, dynamic, and complex environments, where the precision of trajectory control deteriorates over time. The ability to maintain high-precision attitude control under such conditions has thus become a critical research focus in advancing robotic arm technology for practical engineering solutions. The significance of this research lies in its proposed method, which aims to address these challenges by enhancing the robustness and accuracy of robotic arms in real-time operations. By improving control strategies that adapt to changing environmental factors and internal disturbances, this study offers a potential breakthrough in achieving stable, high-precision motion even in unpredictable and complex environments.

To date, numerous control strategies have been proposed to overcome the challenge of industrial manipulator control. Xu et al. [5] introduced an adaptive neural network-based control method for an industrial manipulator. This approach uses neural networks to approximate uncertainties in the control system, with simulations demonstrating effective tracking performance of the system. However, neural networks face some limitations, including long training times and low computational efficiency. To overcome these challenges, Huang et al. [6] proposed an algorithm based on fast recurrent neural networks. This algorithm employs matrix-guided gradient-type recurrent neural networks to efficiently solve optimization problems, significantly enhancing the speed of physical feasibility testing during the dynamic model identification process of the robotic arm. Additionally, Zhou et al. [7] tackled the precise motion control of underwater hydraulic robotic arms by introducing an adaptive robust control method combined with backstepping. This method effectively mitigates the impact of external disturbances, thereby improving the robustness of the controller. Xi et al. [8] proposed a robust adaptive sliding mode controller to address modeling uncertainties and fluctuating disturbances caused by parameter variations during robotic arm operation. This method autonomously adjusts the model parameters, thus improving the robustness of the system. Although these studies comprehensively address the nonlinear characteristics and uncertainties of robotic arm systems, mobile robotic arms in practical applications often face various complex constraints, which makes obtaining precise mathematical models challenging.

Discrete sliding mode control (DSMC) has strong robustness against parameter fluctuations and external disturbances in controlled systems [9]. This is owing to its design principle: guiding the system state trajectory to a pre-set sliding surface and maintaining stable motion of the system state along this surface. This means that once the system state enters the sliding surface, even in the face of partial model changes or external disturbances, the controlled system can still maintain stable operation [10]. For instance, Zhang et al. [11] proposes a novel event-triggered finite-time adaptive sliding mode coordinated control method, which is applied to network-delayed leaf-spring suspension systems to solve the problems of limited bandwidth, spring mass uncertainty, dynamic constraints, and multi-objective control. Zhang et al. [12] proposed a strategy that integrates a Kalman filter, a high-order disturbance observer, and terminal SMC, which effectively solves technical challenges such as model uncertainty, noise, and long sampling intervals in manipulator control. The aforementioned studies have effectively tackled the problem of model uncertainties during robotic arm operation.

Recently, the discrete-time integral sliding mode control (DISMC) method has been widely applied in fields such as disk drives, nuclear reactors, high-speed trains, and buck converters, owing to its significantly smaller tracking error compared to the traditional DSMC method [13,14,15,16,17]. Fu et al. [18] designed a DISMC strategy that considers system uncertainties to address the issue of partial failure in locomotive traction caused by the reduced adhesion performance of heavy-duty trains in severe weather conditions. This strategy achieved precise tracking control of the optimal creep speed. Xu et al. [19] summarized a study where a novel adaptive discrete integral terminal sliding mode control scheme was introduced for manipulator trajectory tracking under measurement disturbances. This scheme achieved precise trajectory tracking with minimal control torque variations, demonstrating its effectiveness and superiority over traditional methods. This method ensured that the tracking error converged within a finite time. However, when facing uncertain random disturbances, the above control strategies may cause a chattering problem.

One effective solution to the chattering problem caused by SMC is adaptive control. Fan et al. [20] proposed an adaptive fast arc tangent nonsingular SMC strategy for an underwater robotic arm. Yao et al. [21] proposed an adaptive robust terminal SMC method for high-speed train operation optimization. This method used an adaptive reaching law to solve the chattering problem. Meanwhile, Xu et al. [22] combined model predictive control (MPC) with DITSMC for a piezoelectric-driven positioning system, achieving motion control under disturbances and suppressing sliding mode chattering. Xu et al. [23] established a discrete-time integral sliding mode predictive control strategy that replaced the adaptive reaching law of DISMC with MPC, achieving higher-precision tracking control.

In summary, this paper presents a new adaptive DITSMPC method for robotic arm dynamic systems operating in complex environments. Firstly, a second-order dynamic model of the manipulator is established by using the Lagrange dynamic strategy. Secondly, a discrete integral terminal sliding mode control law based on an adaptive switching term is designed to achieve high-precision tracking control of the system. Finally, in order to reduce the influence of SMC buffeting on the robot arm system, the prediction time domain function is integrated into the proposed SMC law to estimate the time delay of unknown terms in the robot arm system. The proposed method is designed to provide precise control for robotic arm motion systems under such conditions, taking into account the practical operating scenarios of the robotic arm. The main contributions of this paper are as follows:

(1) A DITSMPC algorithm is proposed, which uses a one-step delay estimation method to compensate for disturbances affecting the controlled system. The method also utilizes an adaptive reaching law and MPC to enhance the trajectory tracking accuracy of the robotic arm and suppress system chattering.
(2) Compared with existing methods that use MPC, neural network control, robust control, etc. [5,6,7,8], this approach does not rely on an exact mathematical model. In contrast to [18,19], this method results in smaller chattering and higher tracking accuracy when dealing with disturbances. Compared to [20,21], it requires fewer tuning parameters and is easier to implement. Compared with [24], this method can be extended to MIMO systems and has stronger applicability.
(3) The main advantage of the proposed DITSMPC scheme is that it provides a control method that is easy to implement and capable of addressing model uncertainties and white noise disturbances in multi-input multi-output systems.

## 2. Mechanical Arm Dynamics Model

A two-degree-of-freedom (2-DOF) robotic arm is a robotic system with two independent movement freedoms, widely used in industrial automation, robotics, education, and research. It typically consists of two rotational joints, where the shoulder joint allows the arm to rotate horizontally and the elbow joint enables rotation in the vertical direction, thus enabling complex movements within a two-dimensional plane. The control of the robotic arm is usually performed through a micro-controller or computer, which drives motors to control the movement of each joint. Its working principle involves both forward kinematics and inverse kinematics—forward kinematics calculates the position of the end effector based on joint angles, while inverse kinematics solves for the required joint angles based on a desired position. The 2-DOF robotic arm is widely used in industrial production for tasks like welding, assembly, and handling, and in the medical field for surgical assistance and rehabilitation training. Additionally, it serves as an educational tool in teaching and robotics research, helping students understand the kinematics and control principles of robotic arms.

### 2.1. Mechanism Model

**Figure 1:**
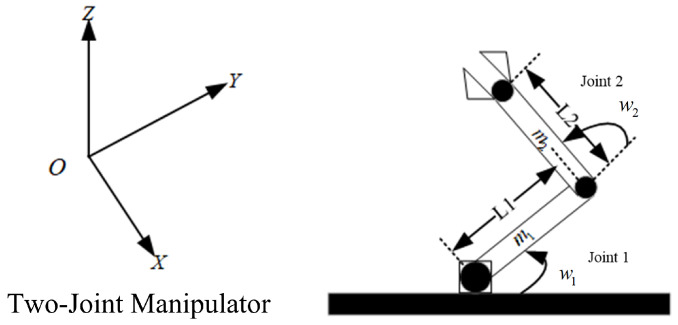
Two-joint manipulator.

Figure 1 shows the dynamic analysis of the two-degree-of-freedom manipulator. Where $L_{1},L_{2},w_{1},w_{2},m_{1}$, and $m_{2}$ denote the lengths of each link, joint angle displacements, and link masses, respectively. Using the Lagrange equation method, the following dynamic model of the system can be obtained.

(1) $$M\left(w\right)\ddot{w}+C\left(w,\dot{w}\right)\dot{w}+G\left(w\right)=ø+d$$

where $M\left(w\right)$, $C\left(w,\dot{w}\right)$, and $G\left(w\right)$ represent the inertia mass matrix, Coriolis matrix, and gravity term matrix, respectively; ø denotes the input; and d represents the uncertainties. $w={\left[\begin{array}{cc} w_{1} & w_{2} \end{array}\right]}^{T}$; $\dot{w}={\left[\begin{array}{cc} {\dot{w}}_{1} & {\dot{w}}_{2} \end{array}\right]}^{T}$; $\ddot{w}={\left[\begin{array}{cc} {\ddot{w}}_{1} & {\ddot{w}}_{2} \end{array}\right]}^{T}$; $ø={\left[\begin{array}{cc} \tau_{1} & \tau_{2} \end{array}\right]}^{T}$; $d={\left[\begin{array}{cc} d_{1} & d_{2} \end{array}\right]}^{T}$.

### 2.2. Discretization of Dynamic Model

The nonlinear dynamic equation of the industrial manipulator can be obtained from Equation (1). Then, we define the state as $s={\left[\begin{array}{cc} w & \dot{w} \end{array}\right]}^{T}$, representing the joint angle displacements and angular velocities of the manipulator; the control input as u=ø, representing the control torque; and the output vector as $y=w^{T}$, representing the joint angle displacement output of the manipulator controlled by the torque. Then, Equation (1) can be equivalent to Equation (2).

(2) $$\left\{\begin{array}{c} \dot{s}=j\left(s,u\right)\\ j\left(s,u\right)=\left[\begin{array}{c} \dot{w}\\ M^{-1}\left(ø-C\left(w,\dot{w}\right)\dot{w}\right) \end{array}\right] \end{array}\right.$$

Using the Jacobian linearization method to deal with Equation (2), the following sub-matrix can be obtained.

(3) $$A_{l}=\left[\begin{array}{cccc} \frac{\partial j_{1}}{\partial s_{1}} & \frac{\partial j_{1}}{\partial s_{2}} & \cdots & \frac{\partial j_{1}}{\partial s_{4}}\\ \frac{\partial j_{2}}{\partial s_{1}} & \frac{\partial j_{2}}{\partial s_{2}} & \cdots & \frac{\partial j_{2}}{\partial s_{4}}\\ \vdots & \vdots & \ddots & \vdots \\ \frac{\partial j_{4}}{\partial s_{1}} & \frac{\partial j_{4}}{\partial s_{2}} & \cdots & \frac{\partial j_{4}}{\partial s_{4}} \end{array}\right]$$

(4) $$B_{l}=\left[\begin{array}{cc} \frac{\partial j_{1}}{\partial u_{1}} & \frac{\partial j_{1}}{\partial u_{2}}\\ \frac{\partial j_{2}}{\partial u_{1}} & \frac{\partial j_{2}}{\partial u_{2}}\\ \vdots & \vdots \\ \frac{\partial j_{4}}{\partial u_{1}} & \frac{\partial j_{4}}{\partial u_{2}} \end{array}\right]$$

Further, Jacobian linearization is performed at the desired equilibrium point. The linearization is carried out at $s=s_{r}$, $\dot{s}={\dot{s}}_{r}$, $u=u_{r}$ using the Jacobian sub-matrices $A_{l},B_{l}$ from Equations (3) and (4), and the following linear equation can be obtained.

(5) $$\dot{s}=A_{l}s+B_{l}u+d$$

By using the zero-order holder and adding the error disturbance term, the following system equation of the MIMO manipulator is obtained:

(6) $$\left\{\begin{array}{c} s(t+1)=\mathrm{As}(t)+\mathrm{Bu}(t)+D(t)\\ y(t)=\mathrm{Cs}(t) \end{array}\right.$$

where $A=e^{A_{l}T_{s}}$; $B=\int_{0}^{T_{s}}e^{A_{l}t}B_{l}\mathrm{dt}$.

We define the disturbance term D(t) as

(7) $$D\left(t\right)={\left[\begin{array}{cccc} d_{1}\left(t\right) & d_{2}\left(t\right) & 0 & 0 \end{array}\right]}^{T}$$

**Assumption 1.**  *There exists an absolute small positive number ι such that Equation (8) holds. The magnitude of ι is an infinitely small quantity of higher-order terms of the system’s sampling time.*

(8) $$\begin{array}{c} d_{i}\left(t\right)=\iota ,\;d_{i}\left(t\right)-d_{i}\left(t-1\right)=2\iota \\ d_{i}\left(t\right)-2d_{i}\left(t-1\right)+d_{i}\left(t-2\right)=3\iota \end{array}$$

## 3. Adaptive Discrete Integral Terminal Sliding Mode Control (ADITSMC) Scheme for Industrial Manipulator

### 3.1. Sliding Mode Control Law

The error of the industrial manipulator can be defined as

(9) $$e\left(t\right)=y\left(t\right)-y_{r}\left(t\right)$$

where $y\left(t\right)$ is the controller output trajectory and $y_{r}\left(t\right)$ is the desired trajectory.

We design the following discrete-time sliding mode function as follows:

(10) $$h\left(t\right)=\lambda_{1}e\left(t\right)+\lambda_{2}F\left(t-1\right)$$

where the integral terminal error term is

(11) $$F\left(t\right)=\sum_{f=0}^{t}{\left(e(f)\right)}^{\alpha }=F\left(t-1\right)+{\left(e(t)\right)}^{\alpha }$$

where $\lambda_{1}>0,\;\lambda_{2}>0,0<\alpha <1$.

Firstly, the following equivalent control $u_{z\mathrm{eq}}\left(t\right)$ condition is given.

(12) Δh(t)=h(t+1)−h(t)=0

where

(13) $$h\left(t+1\right)=\lambda_{1}e\left(t+1\right)+\lambda_{2}F\left(t\right)$$

Further, we obtain

(14) $$\begin{array}{cc} e(t+1) & =y\left(t+1\right)-y_{r}\left(t+1\right)\\ & =\mathrm{CAs}\left(t\right)+\mathrm{CBu}\left(t\right)-\mathrm{CD}\left(t\right)-y_{r}\left(t+1\right) \end{array}$$

Furthermore, the following disturbance delay estimation strategy is designed.

(15) D(t−1)=s(t)−As(t−1)−Bu(t−1)

Combining Equations (6) and (9)–(15), the following ADITSMC law for the manipulator can be obtained:

(16) $$\begin{array}{c} u_{zeq}\left(t\right)={\left(\mathrm{CB}\right)}^{-1}\left[\lambda_{1}^{-1}\right.h\left(t\right)-\lambda_{1}^{-1}\lambda_{2}F\left(t\right)+y_{r}\left(t+1\right)-\mathrm{CAs}\left(t\right)-\left.\mathrm{CD}(t-1)\right] \end{array}$$

Based on the equivalent control, the following nonlinear switching control law is further introduced.

(17) $$u_{sw}\left(t\right)=-{\left(\mathrm{CB}\right)}^{-1}\beta \mathrm{sgn}\left(h(t)\right)$$

where β=2ι.

Furthermore, the following parameter-adaptive law is designed.

(18) $$\begin{array}{cc} {\hat{\beta }}_{i}\left(t\right)=\frac{T_{s}}{\varsigma }\left|h_{i}\left(t\right)\right| & i=1,2,3,\cdots ,n \end{array}$$

where ς is a constant.

In summary, the ADITSMC law for the manipulator can be obtained

(19) $$\begin{array}{c} u_{zeq}\left(t\right)={\left(\mathrm{CB}\right)}^{-1}\left[\lambda_{1}^{-1}\right.h\left(t\right)-\lambda_{1}^{-1}\lambda_{2}F\left(t\right)+y_{r}\left(t+1\right)-\mathrm{CAs}\left(t\right)-\mathrm{CD}\left(t-1\right)-\left.J(t)\right] \end{array}$$

where

(20) $$J\left(t\right)=\left[\begin{array}{c} {\hat{\beta }}_{1}\left(t\right)\mathrm{sgn}\left(h_{1}\left(t\right)\right)\\ {\hat{\beta }}_{2}\left(t\right)\mathrm{sgn}\left(h_{2}\left(t\right)\right)\\ \vdots \\ {\hat{\beta }}_{n}\left(t\right)\mathrm{sgn}\left(h_{n}\left(t\right)\right) \end{array}\right]$$

### 3.2. Proof of Convergence

Substituting Equation (19) into Equation (14), we have:

(21) $$e\left(t+1\right)=\lambda_{1}^{-1}h\left(t\right)-\lambda_{1}^{-1}\lambda_{2}F\left(t\right)-J\left(t\right)+C\left[D(t)-D(t-1)\right]$$

By substituting Equation (21) into Equation (10), we can derive

(22) $$h\left(t+1\right)=h\left(t\right)-J\left(t\right)+C\left[D(t)-D(t-1)\right]$$

We define

(23) $$G\left(t\right)=C\left[D(t)-D(t-1)\right]$$

For MIMO systems, the matrices F(t) and h(t) can be represented as

(24) $$G\left(t\right)\overset{\Delta }{=}{\left[G_{1}\left(t\right)\cdots G_{n}\left(t\right)\right]}^{T}$$

(25) $$h\left(t\right)\overset{\Delta }{=}{\left[h_{1}\left(t\right)\cdots h_{n}\left(t\right)\right]}^{T}$$

According to Assumption 1, there must exist a sufficiently small number κ such that Equation (26) holds.

(26) $${\left|G_{i}\left(t\right)\right|}_{\infty }\leq \kappa$$

Taking $\beta_{i}\left(t\right)>\beta >\kappa$ and rewriting Equation (22) in terms of the matrix sub-elements, we obtain

(27) $$h_{i}\left(t+1\right)=h_{i}\left(t\right)-\beta \mathrm{sgn}\left(h_{i}\left(t\right)\right)+G_{i}\left(t\right)$$

The reaching conditions [10] of SMC are as follows:

(28) $$\left\{\begin{array}{c} \left[h_{i}\left(t+1\right)-h_{i}\left(t\right)\right]\mathrm{sgn}\left(h_{i}\left(t\right)\right)<0\\ \left[h_{i}\left(t+1\right)+h_{i}\left(t\right)\right]\mathrm{sgn}\left(h_{i}\left(t\right)\right)>0 \end{array}\right.$$

When system (6) is not within the SMC manifold, we have

(29) $$\begin{array}{c} \left[h_{i}\left(t+1\right)-h_{i}\left(t\right)\right]\mathrm{sgn}\left(h_{i}\left(t\right)\right)\\ =\left[G_{i}\left(t\right)-\beta \mathrm{sgn}\left(h_{i}\left(t\right)\right)\right]\mathrm{sgn}\left(h_{i}\left(t\right)\right)\\ =G_{i}\left(t\right)\mathrm{sgn}\left(h_{i}\left(t\right)\right)-\beta \\ <\kappa -\beta \\ <0 \end{array}$$

(30) $$\begin{array}{c} \left[h_{i}\left(t+1\right)+h_{i}\left(t\right)\right]\mathrm{sgn}\left(h_{i}\left(t\right)\right)\\ =\left[2h_{i}\left(t\right)+G_{i}\left(t\right)-\beta \mathrm{sgn}\left(h_{i}\left(t\right)\right)\right]\mathrm{sgn}\left(h_{i}\left(t\right)\right)\\ =2\left|h_{i}\left(t\right)\right|+G_{i}\left(t\right)\mathrm{sgn}\left(h_{i}\left(t\right)\right)-\beta \\ >\kappa +\beta \\ >0 \end{array}$$

From Equations (28)–(30), it can be determined that the proposed scheme satisfies the sliding mode arrival condition.

Further, it is necessary to obtain the quasi-sliding mode bandwidth of the proposed scheme.

The Lyapunov function is designed as follows:

(31) $$V_{i}\left(t\right)=h_{i}^{2}\left(t\right)$$

By taking the derivative of the Lyapunov function, we obtain

(32) $${\dot{V}}_{i}\left(t\right)=\frac{\Delta V_{i}\left(t\right)}{T}$$

Since T>0 is always true, the signs of ${\dot{V}}_{i}\left(t\right)$ and $\Delta V_{i}\left(t\right)$ are equal. Hence, it is proven that $\Delta V_{i}\left(t\right)<0$. This will be divided into two conditions:

(1) Case 1: $h_{i}\left(t\right)>\kappa +\beta$

(33) $$\begin{array}{c} \Delta V_{i}\left(t\right)=V_{i}\left(t+1\right)-V_{i}\left(t\right)\\ \;=h_{i}^{2}\left(t+1\right)-h_{i}^{2}\left(t\right)\\ \;=\left[h_{i}\left(t+1\right)-h_{i}\left(t\right)\right]\left[h_{i}\left(t+1\right)+hh_{i}\left(t\right)\right]\\ \;=\left[G_{i}\left(t\right)-\beta \mathrm{sgn}\left(h_{i}\left(t\right)\right)\right]\left[2h_{i}\left(t\right)+G_{i}\left(t\right)-\beta \mathrm{sgn}\left(h_{i}\left(t\right)\right)\right]\\ \;=\left[G_{i}\left(t\right)-\beta \right]\left[2h_{i}\left(t\right)+G_{i}\left(t\right)-\beta \right] \end{array}$$

where

(34) $$-\kappa -\beta <G_{i}\left(t\right)-\beta <\kappa -\beta <0$$

and

(35) $$2h_{i}\left(t\right)+G_{i}\left(t\right)-\beta >\kappa +\beta >0$$

Hence, we obtain

(36) $$\Delta V_{i}\left(t\right)<\left(\kappa -\beta \right)\left(\kappa +\beta \right)<\kappa^{2}-\beta^{2}<0$$

Because

(37) $$\Delta V_{i}\left(t\right)=h_{i}^{2}\left(r+1\right)-h_{i}^{2}\left(r\right)\leq \kappa^{2}-\beta^{2}$$

Based on Equation (37), Equation (38) can be derived.

(38) $$\begin{array}{c} h_{i}^{2}\left(1\right)\leq h_{i}^{2}\left(0\right)+\left(\kappa^{2}-\beta^{2}\right)\\ h_{i}^{2}\left(2\right)\leq h_{i}^{2}\left(0\right)+2\left(\kappa^{2}-\beta^{2}\right)\\ h_{i}^{2}\left(3\right)\leq h_{i}^{2}\left(0\right)+3\left(\kappa^{2}-\beta^{2}\right)\\ \vdots \\ h_{i}^{2}\left(r_{i}\right)\leq h_{i}^{2}\left(0\right)+r_{i}\left(\kappa^{2}-\beta^{2}\right) \end{array}$$

When $h_{i}^{2}\left(0\right)+{\hat{r}}_{i}\left(\kappa^{2}-\beta^{2}\right)={\left(\kappa +\beta \right)}^{2}$, it can be deduced that $h_{i}^{2}\left({\hat{r}}_{i}\right)\leq {\left(\kappa +\beta \right)}^{2}$. This indicates that after taking an integer number of steps determined by ${\hat{r}}_{i}$, system (6) will enter a quasi-sliding mode centered on the sliding mode switching surface, with ε=κ+β serving as the bandwidth for the quasi-sliding mode.

Based on Equation (38), the relevant solution (39) can be obtained.

(39) $${\hat{r}}_{i}=\frac{h_{i}\left(0\right)-\leq {\left(\kappa +\beta \right)}^{2}}{\beta^{2}-\kappa^{2}}$$

We define $\left|{\hat{r}}_{i}\right|$ as the greatest integer less than or equal to $\left|{\hat{r}}_{i}\right|$.

(40) $$t=\max_{1\leq i\leq n}\left\{\left|\frac{h_{i}\left(0\right)-\leq {\left(\kappa +\beta \right)}^{2}}{\beta^{2}-\kappa^{2}}\right|\right\}+1$$

Therefore, it can be concluded that the quasi-sliding mode bandwidth of the system is ε=κ+β.

(2) Case 2: $h_{i}\left(t\right)<-\kappa -\beta$. Similarly, it can be deduced that

(41) $$\Delta V_{i}\left(t\right)=\left[G_{i}\left(t\right)+\beta \right]\left[2h_{i}\left(t\right)+G_{i}\left(t\right)+\beta \right]$$

where

(42) $$0<-\kappa +\beta <G_{i}\left(t\right)+\beta <\kappa +\beta$$

and

(43) $$2h_{i}\left(t\right)+G_{i}\left(t\right)+\beta <-\kappa -\beta <0$$

Hence,

(44) $$\Delta V_{i}\left(t\right)<\left(-\kappa -\beta \right)\left(-\kappa +\beta \right)<\kappa^{2}-\beta^{2}<0$$

because

(45) $$\Delta V_{i}\left(t\right)=h_{i}^{2}\left(r+1\right)-h_{i}^{2}\left(r\right)\leq \kappa^{2}-\beta^{2}$$

Since the expression for $\Delta V_{i}\left(t\right)$ is the same, it can similarly be deduced that $h_{i}^{2}\left({\hat{r}}_{i}\right)\leq {\left(\kappa +\beta \right)}^{2}$. This means that after a certain number of integer steps determined by rounding ${\hat{r}}_{i}$, system (6) will enter a quasi-sliding mode centered on the sliding mode switching surface, with ε=κ+β serving as the bandwidth for the quasi-sliding mode.

Similarly, it can be deduced that

(46) $${\hat{r}}_{i}=\frac{h_{i}\left(0\right)-\leq {\left(\kappa +\beta \right)}^{2}}{\beta^{2}-\kappa^{2}}$$

We define $\left|{\hat{r}}_{i}\right|$ as the greatest integer less than or equal to ${\hat{r}}_{i}$.

(47) $$\begin{array}{cc} k= & \max_{1\leq i\leq n}\left\{\left|{\hat{r}}_{i}\right|\right\}+1\\ = & \max_{1\leq i\leq n}\left\{\left|\frac{h_{i}\left(0\right)-\leq {\left(\kappa +\beta \right)}^{2}}{\beta^{2}-\kappa^{2}}\right|\right\}+1 \end{array}$$

According to (47), the convergence of the designed ADITSMC is rigorously proven.

Therefore, regardless of the initial state, system (6) will reach the switching surface within a finite number of steps and then move along it. However, its motion can be divided into three stages: the approaching mode, quasi-sliding mode, and steady state. Therefore, the final step is to prove that once system (6) enters the switching surface, it will not escape from it.

Next, we define a switching band that surrounds the switching surface:

(48) $$\Theta =\left\{h\left(t\right):\left|h_{i}\left(t\right)\leq \kappa +\beta =2\iota \right|\right\}$$

Then, we will analyze the following two scenarios:

(1) Case 1: $0\leq h_{i}\left(t\right)\leq \kappa +\beta$

(49) $$h_{i}\left(t+1\right)=h_{i}\left(t\right)-\beta \mathrm{sgn}\left(h_{i}\left(t\right)\right)+G_{i}\left(t\right)$$

Based on Equation (49), we have

(50) $$-\kappa -\beta <h_{i}\left(t+1\right)<2\kappa <\kappa +\beta$$

(2) Case 2: $-\kappa -\beta \leq h_{i}\left(t\right)\leq 0$

(51) $$h_{i}\left(t+1\right)=h_{i}\left(t\right)-\beta \mathrm{sgn}\left(h_{i}\left(t\right)\right)+G_{i}\left(t\right)$$

From Equation (51), we can derive

(52) $$-\kappa -\beta <-2\kappa <h_{i}\left(t+1\right)<\kappa +\beta$$

Based on Equations (49)–(53) we can draw the conclusion that once system (6) enters the region defined by Equation (48), it will never escape from that region. Here, ε=κ+β denotes the bandwidth of the quasi-sliding mode, with a magnitude of 2ι.

Next, we will prove that the designed adaptive discrete integral terminal sliding mode control is stable and can enter the quasi-sliding mode in a finite number of steps.

Given that the system bandwidth ε=κ+β=2ι, it implies that $\left|h_{i}\left(t\right)\right|$ is bounded. Therefore, as long as Equation (53) is satisfied, system (6) will be stable and can enter the quasi-sliding mode in a finite number of steps.

(53) $$\begin{array}{ccc} \beta_{i}\left(t\right)>\beta >\kappa & \Rightarrow & \alpha <\frac{T_{s}\left|h_{i}\left(t\right)\right|}{\kappa } \end{array}$$

In summary, system (6) satisfies the discrete sliding mode reaching condition and will enter a quasi-sliding mode centered on the sliding mode switching surface, with ε=κ+β serving as the bandwidth for the quasi-sliding mode, in a finite number of steps.

## 4. Design of DITSMPC Scheme

In the DITSMPC law for robotic manipulators, the MPC method is employed to replace the switching control law $u_{mp}$, and an optimal control law is generated. This control law drives system (6) to the sliding surface, and then maintains system (6) on the sliding surface through the equivalent control law $u_{zeq}$ derived in the previous section, thereby achieving the objective of attitude tracking control for the robotic manipulator system.

### 4.1. DITSMPC Law

The following composite control law is designed:

(54) $$U\left(t\right)=u_{zeq}\left(t\right)+u_{mp}\left(t\right)$$

Substituting Equation (54) into Equation (14) yields Equation (55).

(55) $$\begin{array}{cc} e(t+1) & =y\left(t+1\right)-y_{r}\left(t+1\right)\\ & =\mathrm{CAs}\left(t\right)+\mathrm{CB}u_{zeq}\left(t\right)+\mathrm{CB}u_{mp}\left(t\right)+\mathrm{CD}\left(t\right)-y_{r}\left(t+1\right) \end{array}$$

Substituting Equation (55) into Equation (13) yields Equation (56).

(56) $$h\left(t+1\right)=h\left(t\right)+\lambda_{1}\mathrm{CB}u_{mp}\left(t\right)-\lambda_{1}G\left(t\right)$$

where

(57) $$G\left(t\right)=C\left[D(t)-D(t-1)\right]$$

By recursively applying Equation (56), we can obtain Equation (58).

(58) $$\begin{array}{cc} h(t+2) & =h\left(t+1\right)+\lambda_{1}\mathrm{CB}u_{mp}\left(t+1\right)+\lambda_{1}G\left(t+1\right)\\ & =h\left(t\right)+\lambda_{1}\mathrm{CB}\left(u_{mp}\left(t\right)+u_{mp}\left(t+1\right)\right)+\lambda_{1}\left(G(t)+G(t+1)\right)\\ & \vdots \\ h(t+N) & =h\left(t\right)+\lambda_{1}\mathrm{CB}\left(u_{mp}\left(t\right)\right.+u_{mp}\left(t+1\right)+\cdots +u_{mp}\left.\left(t+N-1\right)\right)\\ & +\lambda_{1}\left(G\right.\left(t\right)+G\left(t+1\right)+\cdots +G\left.\left(t+N-1\right)\right) \end{array}$$

We rewrite Equations (56) and (58) in matrix form, as shown in Equation (59).

(59) $$\begin{array}{c} \left[\begin{array}{c} h(t+1)\\ h(t+2)\\ \vdots \\ h(t+N) \end{array}\right]=\left[\begin{array}{c} I\\ I\\ \vdots \\ I \end{array}\right]\cdot h\left(t\right)+\\ \left[\begin{array}{c} \lambda_{1}\mathrm{CB}0\cdots 0\\ \lambda_{1}\mathrm{CB}\lambda_{1}\mathrm{CB}\cdots 0\\ \vdots \vdots \ddots \vdots \\ \lambda_{1}\mathrm{CB}\lambda_{1}\mathrm{CB}\cdots \lambda_{1}\mathrm{CB} \end{array}\right]\cdot \left[\begin{array}{c} u_{mp}\left(t\right)\\ u_{mp}\left(t+1\right)\\ \vdots \\ u_{mp}\left(t+1\right) \end{array}\right]+\\ \left[\begin{array}{c} \lambda_{1}0\cdots 0\\ \lambda_{1}\lambda_{1}\cdots 0\\ \vdots \vdots \ddots \vdots \\ \lambda_{1}\lambda_{1}\cdots \lambda_{1} \end{array}\right]\cdot \left[\begin{array}{c} G(t)\\ G(t+1)\\ \vdots \\ G(t+N-1) \end{array}\right] \end{array}$$

Let

(60) $$H\left(t\right)={\left[\begin{array}{cccc} h(t+1) & h(t+2) & \cdots & h(t+N) \end{array}\right]}^{T}$$

(61) $$U\left(t-1\right)={\left[\begin{array}{ccc} u_{mp}\left(t\right) & \cdots & u_{mp}\left(t+N-1\right) \end{array}\right]}^{T}$$

(62) $$ı\left(t-1\right)={\left[\begin{array}{cccc} G(t) & G(t+1) & \cdots & G(t+N-1) \end{array}\right]}^{T}$$

(63) $$\mathrm{ffi}={\left[\begin{array}{cccc} I & I & \cdots & I \end{array}\right]}^{T}$$

(64) $$\Phi =\left[\begin{array}{cccc} \lambda_{1}\mathrm{CB} & 0 & \cdots & 0\\ \lambda_{1}\mathrm{CB} & \lambda_{1}\mathrm{CB} & \cdots & 0\\ \vdots & \vdots & \ddots & \vdots \\ \lambda_{1}\mathrm{CB} & \lambda_{1}\mathrm{CB} & \cdots & \lambda_{1}\mathrm{CB} \end{array}\right]$$

(65) $$œ=\left[\begin{array}{cccc} \lambda_{1}I & 0 & \cdots & 0\\ \lambda_{1}I & \lambda_{1}I & \cdots & 0\\ \vdots & \vdots & \ddots & \vdots \\ \lambda_{1}I & \lambda_{1}I & \cdots & \lambda_{1}I \end{array}\right]$$

Based on Equation (59) to Equation (65), we can derive Equation (66).

(66) H(t)=ffih(t)+ΦU(t−1)+œı(t−1)

To obtain the optimal control for the robotic manipulator, the following performance index function is introduced:

(67) $$J=H^{T}\left(t\right)H\left(t\right)+YU^{T}\left(t-1\right)U\left(t-1\right)$$

where Y denotes the weighting parameter, and its value determines the magnitude of the control input. To minimize the performance index function, we take its partial derivative and set it equal to zero.

(68) $$\frac{\partial J}{\partial U(t-1)}=0$$

Then, by substituting Equation (66) into Equation (67), the performance index function is transformed into Equation (69).

(69) $$\Phi^{T}\left[\mathrm{ffi}h(t)+\Phi U(t-1)+œı(t-1)\right]=-YU\left(t-1\right)$$

Based on Equation (69), we can solve for Equation (70).

(70) $$U\left(t-1\right)=-{\left(\Phi^{T}\Phi +YI\right)}^{-1}\Phi^{T}\left[\mathrm{ffi}h(t)+œı(t-1)\right]$$

For this system, the future values of the disturbance estimation errors are unknown. Therefore, we use the disturbance estimation errors from the previous time step to replace them. Based on this, we can obtain Equation (71).

(71) $$G\left(t-1\right)=C\left[D\left(t-1\right)-\hat{D}\left(t-1\right)\right]$$

Similarly to the previous section, $\hat{D}\left(t-1\right)$ is estimated based on the previous time step and can be obtained using the following equation.

(72) $$\hat{D}\left(t\right)=D\left(t-1\right)=s\left(t\right)-\mathrm{As}\left(t-1\right)-\mathrm{BU}\left(t-1\right)$$

Therefore, Equation (62) is transformed into Equation (73).

(73) $$\hat{ı}\left(t-1\right)={\left[\begin{array}{cccc} G(t-1) & G(t-1) & \cdots & \;G(t-1) \end{array}\right]}^{T}$$

Considering that only the first element of the predictive control sequence is used, by combining Equations (70) to (73), the optimal control law for the system can be obtained as follows:

(74) $$\begin{array}{cc} u_{mp}\left(t\right) & =\mathrm{VU}(t-1)\\ & =-V{\left(\Phi^{T}\Phi +YI\right)}^{-1}\Phi^{T}\left[\mathrm{ffi}h\left(t\right)+œ\hat{ı}\left(t-1\right)\right] \end{array}$$

where

(75) $$V=\left[\begin{array}{cccc} I & 0 & \cdots & 0 \end{array}\right]$$

**Figure 2:**
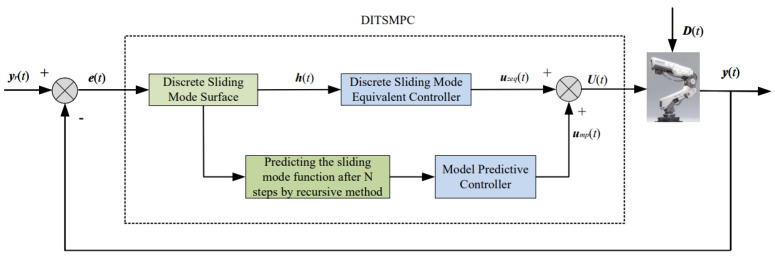
DITSMPC for second-order manipulator.

By combining Equations (19), (54), and (74), the total control law for the system can be obtained as shown in Equation (76), and its control block diagram is shown in Figure 2.

(76) $$\begin{array}{cc} U(t)= & {\left(\mathrm{CB}\right)}^{-1}\left(\lambda_{1}^{-1}\right.h\left(t\right)-\lambda_{1}^{-1}\lambda_{2}G\left(t\right)+y_{r}\left(t+1\right)-\mathrm{CAs}\left(t\right)\\ - & \left.\mathrm{CD}(t-1)\right)-V{\left(\Phi^{T}\Phi +YI\right)}^{-1}\Phi^{T}\left[\mathrm{ffi}h\left(t\right)+œ\hat{ı}\left(t-1\right)\right] \end{array}$$

### 4.2. Proof of Convergence

After substituting Equation (70) into Equation (76), we can obtain Equation (77).

(77) $$H\left(t\right)=\mathrm{ffi}h\left(t\right)-\Phi {\left(\Phi^{T}\Phi +YI\right)}^{-1}\Phi^{T}\left[\mathrm{ffi}h\left(t\right)+œ\hat{ı}\left(t-1\right)\right]+œı\left(t-1\right)$$

The weighting parameter Y defined in Equation (67) is to limit the control action of MPC. However, for steady-state analysis, transient switching control actions do not need to be considered, i.e., we set Y=0. At this point, Equation (77) is transformed into Equation (78).

(78) $$H\left(t\right)=œı\left(t-1\right)-œ\hat{ı}\left(t-1\right)$$

Since only the first element of the predictive control sequence is used, Equation (78) is transformed into Equation (79).

(79) $$h\left(t+1\right)=\lambda_{1}\left[G(t)-G(t-1)\right]$$

By combining Equations (57), (71), (72), and (79), we can obtain Equation (80).

(80) $$\begin{array}{cc} h(t+1) & =\lambda_{1}C\left[D(t)-D(t-1)-D(t-1)-D(t-2)\right]\\ & =\lambda_{1}C\left[D(t)-2D(t-1)-D(t-2)\right]\\ & =3\iota \end{array}$$

Based on Assumption 1, we know that the magnitude of h(t+1) is in the order of 3ι. Therefore, according to Equation (80), the limit of h(t+1) is bounded. Consequently, from Equation (80), we can derive Equation (81).

(81) $$h_{i}\left(t+1\right)\leq \varepsilon =3\iota ,\;i=1,2,\cdots ,n$$

To sum up, we can conclude that system (6) is stable.

## 5. Simulation Experiment

### 5.1. Two-Joint Manipulator

**Figure 3:**
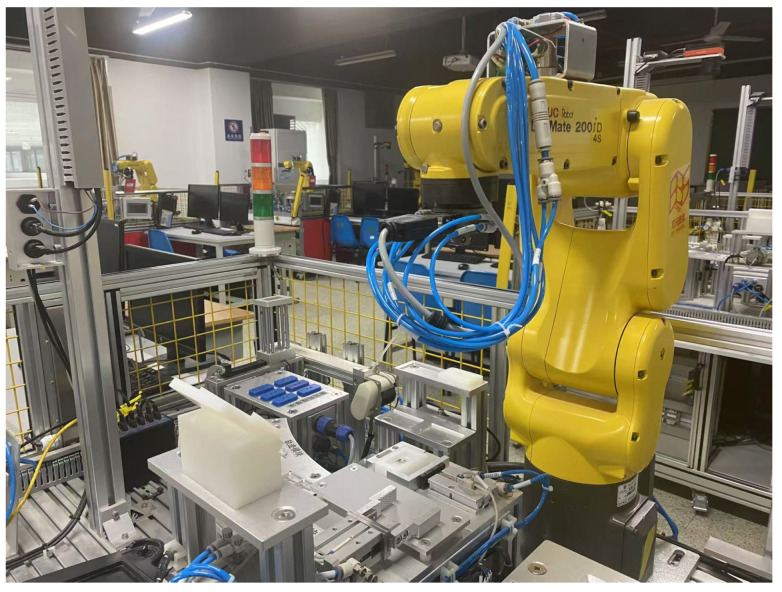
Two-joint manipulator.

The 2-DOF manipulator is a fundamental and significant robotic system widely used in education, experimentation, and basic industrial tasks. It comprises a fixed base, a shoulder joint, an elbow joint, linkages, an end effector (e.g., gripper), and a control system. The physical structure of the manipulator is shown in Figure 3. Capable of moving along two axes within a 2D plane, it precisely executes straightforward automation tasks. Due to its simple structure and limited DOFs, it serves as an ideal educational platform, extensively used in universities and labs to teach robotics fundamentals. In industrial applications, while not as versatile as complex systems, it offers a cost-effective solution for specific tasks like pick-and-place, basic assembly, and material handling. It is easily integrated into automated production lines, especially for small-scale or prototype manufacturing. Despite its limited mobility, its ability to perform precise movements in controlled environments makes it a valuable resource for learning and experimentation.

### 5.2. Simulation Parameter Settings

In the simulation process, the designed DITSMPC method is employed for trajectory tracking control. This control method combines the advantages of SMC and MPC, and can effectively deal with the external disturbance and the uncertainty of system parameters. Firstly, the system model information is given as follows:

(82) $$M\left(w\right)\left[\begin{array}{c} {\ddot{w}}_{1}\\ {\ddot{w}}_{2} \end{array}\right]+C\left(w,\dot{w}\right)\left[\begin{array}{c} {\dot{w}}_{1}\\ {\dot{w}}_{2} \end{array}\right]+G\left(w\right)=\left[\begin{array}{c} \tau_{1}\\ \tau_{2} \end{array}\right]+\left[\begin{array}{c} d_{1}\\ d_{2} \end{array}\right].$$

The parameters in the equation are shown as in Equations (83)–(85).

(83) $$M\left(w\right)=\left[\begin{array}{cc} v+w_{01}+w_{02}\cos\left(w_{2}\right) & w_{01}+w_{02}\cos\left(w_{2}\right)\\ w_{01}+w_{02}\cos\left(w_{2}\right) & w_{01} \end{array}\right],$$

(84) $$C\left(w,\dot{w}\right)=\left[\begin{array}{cc} -w_{02}{\dot{w}}_{2}\sin\left(w_{2}\right) & -w_{02}\left({\dot{w}}_{1}+{\dot{w}}_{2}\right)\sin\left(w_{2}\right)\\ w_{02}{\dot{w}}_{1}\sin\left(w_{2}\right) & 0 \end{array}\right],$$

(85) $$G\left(w\right)=\left[\begin{array}{c} 15g\cos w_{1}+8.75g\cos\left(w_{1}+w_{2}\right)\\ 8.75g\cos\left(w_{1}+w_{2}\right) \end{array}\right].$$

where v=13.33, $w_{01}=8.98$, $w_{02}=8.75$, g=9.8. The system is discretized using the sampling time $T_{s}=0.01s$.

Under the same experimental conditions, the control effects of three strategies are compared: DITSMPC, proposed in Section 4; DITSMC, proposed in [25,26]; and ADITSMC, proposed in Section 3. The simulation parameters are detailed in Table 1.

### 5.3. Simulation Results

**Figure 4:**
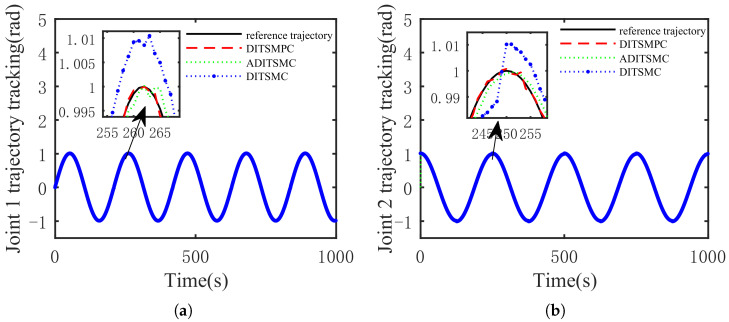
The difference in the control effect under the three methods (tracking). (**a**) Joint 1 tracking, (**b**) Joint 2 tracking.

**Figure 5:**
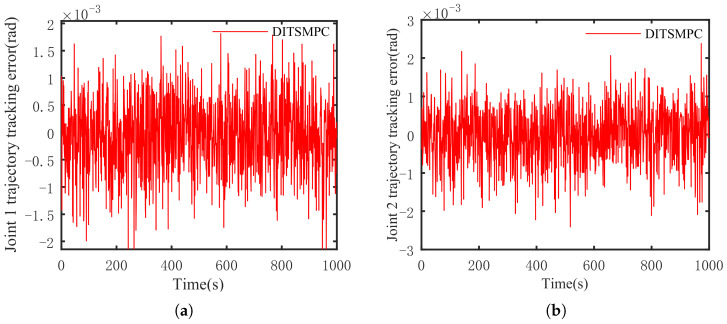
The trajectory tracking error of DITSMPC. (**a**) Joint 1, (**b**) Joint 2.

**Figure 6:**
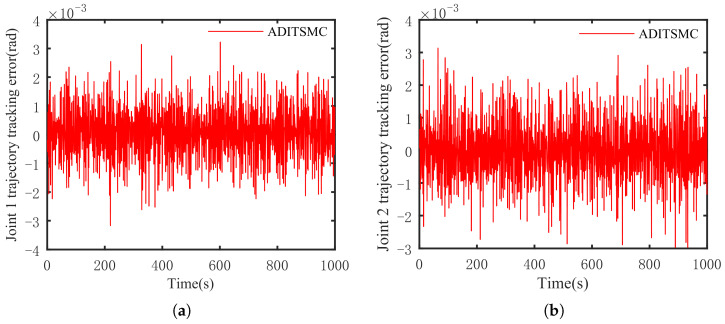
The trajectory tracking error of ADITSMC. (**a**) Joint 1, (**b**) Joint 2.

**Figure 7:**
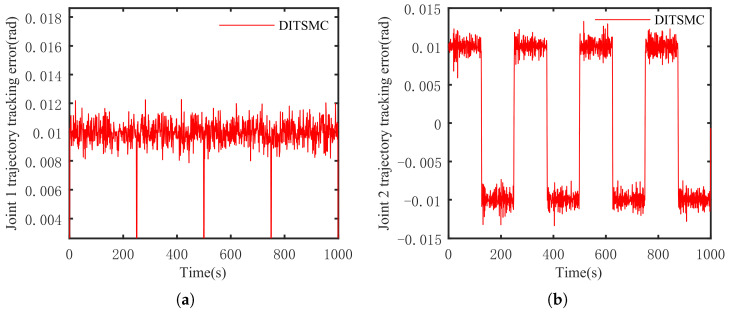
The trajectory tracking error of DITSMC. (**a**) Joint 1, (**b**) Joint 2.

**Figure 8:**
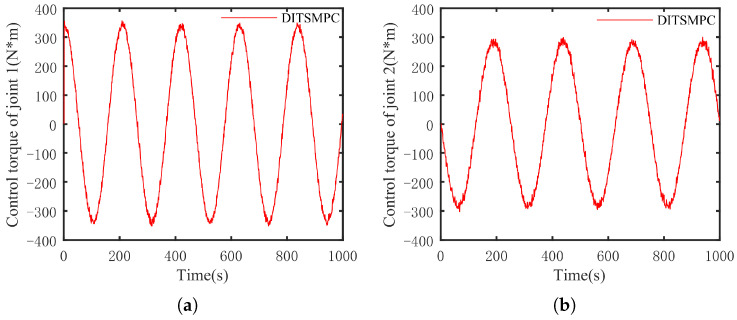
The control input of DITSMPC. (**a**) Joint 1, (**b**) Joint 2.

**Figure 9:**
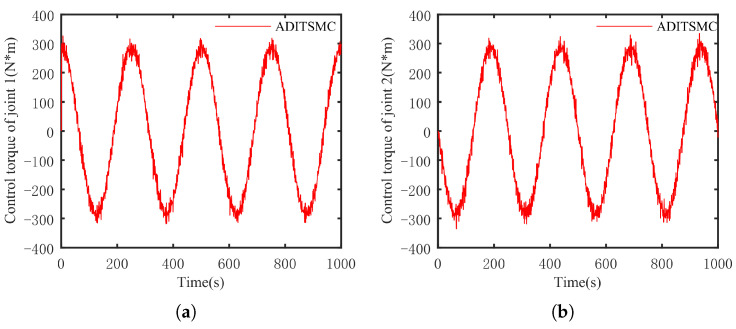
The control input of ADITSMC. (**a**) Joint 1, (**b**) Joint 2.

**Figure 10:**
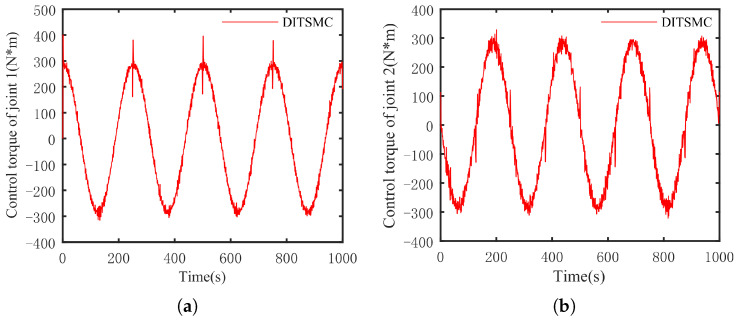
The control input of DITSMC. (**a**) Joint 1, (**b**) Joint 2.

The optimal simulation results under the criteria outlined in Section 5.2 were as follows. The tracking capabilities of the various manipulator joints under the DITSMPC, ADITSMC, and DITSMC approaches are shown in Figure 4, respectively. The tracking errors of the various robotic arm joints for the three control systems are shown in Figure 5, Figure 6 and Figure 7. The inputs for the various joints under different comparison schemes are shown in Figure 8, Figure 9 and Figure 10.

According to the Figure 4, it is evident that for Joint 1, the DITSMPC approach exhibits superior control performance compared to the ADITSMC approach, while the DITSMC approach performs worse than both of the aforementioned methods. Specifically, within the time interval of [255 s, 265 s], the trajectory of the DITSMPC method aligns more closely with the reference trajectory, demonstrating better control effectiveness compared to ADITSMC and DITSMC. This trend is also apparent in Joint 2, where, during the time interval of [245 s, 255 s], the DITSMPC method continues to outperform the other two control strategies. Conversely, the DITSMC approach shows stepped trajectory variations, which are not permissible in practical engineering applications.

Figure 5, Figure 6 and Figure 7 show that the DITSMPC technique exhibits the best control performance for both Joints 1 and 2 of the robotic arm, with the fewest tracking errors and no step changes, demonstrating its better disturbance rejection capacity. Even though the ADITSMC technique performs less well than DITSMPC in the face of white noise interference, it nevertheless exhibits respectable performance. In contrast, the DITSMC approach exhibits significantly larger trajectory tracking errors and noticeable oscillations. Specifically, for the DITSMC approach, the tracking error for Joint 1 remains stable within the range of [0.008, 0.019 rad], but oscillates severely. In Joint 2, where the tracking error varies about zero, this problem is more noticeable and makes the robotic arm unsuitable for real-world engineering applications.

The suggested DITSMPC method, on the other hand, shows no variation in control performance. It demonstrates rapid convergence, robust interference attenuation capabilities, and excellent control performance by achieving trajectory tracking errors for two joints that converge within [−0.002 rad, 0.002 rad], even under white noise interference.

Based on Figure 8, Figure 9 and Figure 10, the DITSMC approach exhibits a step-like phenomenon when the trajectory direction of the robotic arm changes. This behavior is attributed to the invariant parameters of the SMC surface reaching law. The ADITSMC approach, as designed in this work, shows less variation in control torques for both Joint 1 and Joint 2 under white noise interference compared to the DITSMC approach. Moreover, no oscillations occur during trajectory transitions with the ADITSMC approach. Although the differences between the DITSMPC and ADITSMC approaches are not prominently discernible in the figures, this paper employs two metrics—the Mean Squared Error (MSE) and Integral of Absolute Force Variation (IAFV)—to quantitatively evaluate the proposed control method.

(1) MSE
  (86) $$\mathrm{MSE}=\frac{1}{nT_{t}}\sum_{i=1}^{n}\sum_{t=1}^{T_{t}}{\left|e_{i}\left(t\right)\right|}^{2},$$
(2) IAFV
  (87) $$\mathrm{IAFV}=\sum_{i=1}^{n}\sum_{t=2}^{T_{t}}\left|u_{i}\left(t\right)-u_{i}\left(t-1\right)\right|.$$

Table 2 displays the outcomes of the two performance measures’ computations for every control strategy. As observed from the MSE, the DITSMPC method achieves the smallest MSE, indicating the best tracking performance, followed by ADITSMC, and the DITSMC method exhibits a step-like phenomenon when the robotic arm’s trajectory direction changes, leading to a slightly larger MSE. This trend is similarly reflected in the IAFV results.

Lastly, the trajectory tracking performance of the suggested control strategy is confirmed when the robotic arm encounters increasingly complicated trajectories. The two axes’ intended trajectories are fictitiously set to $3\times \sin\left(0.02t\right)+2\times \cos\left(0.097t\right)$ during the simulation.

**Figure 11:**
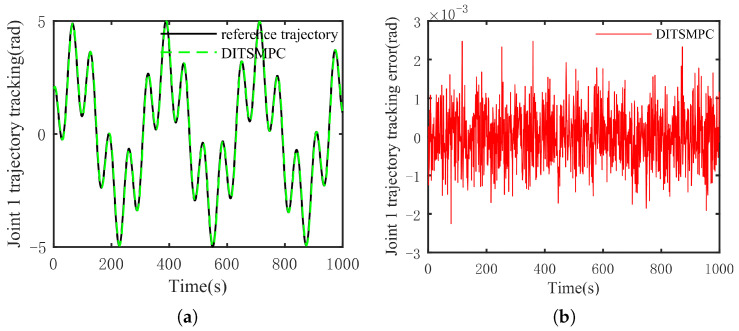
The tracking effect of the DITSMPC method and its error for Joint 1 under complex target trajectory conditions. (**a**) Tracking situation, (**b**) error.

**Figure 12:**
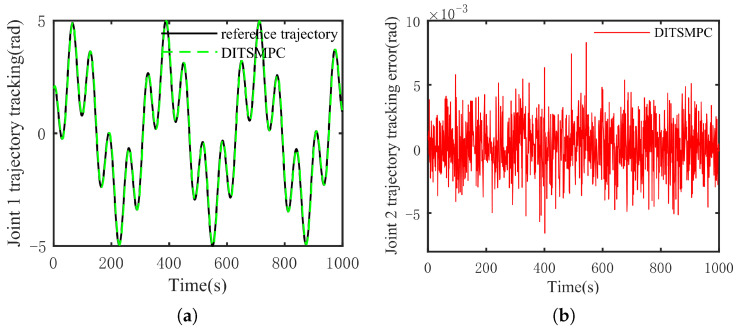
The tracking effect of the DITSMPC method and its error for Joint 2 under complex target trajectory conditions. (**a**) Tracking situation, (**b**) error.

Figure 11 and Figure 12 demonstrate that DITSMPC can track the desired trajectory with negligible errors even when confronted with more complicated trajectories. Despite regular changes in trajectory direction, there are no notable abrupt changes in the magnitude of errors. Consequently, it can be said that even when system (6) is presented with more complicated trajectories, the method suggested in this study can still steadily track the desired trajectory.

**Figure 13:**
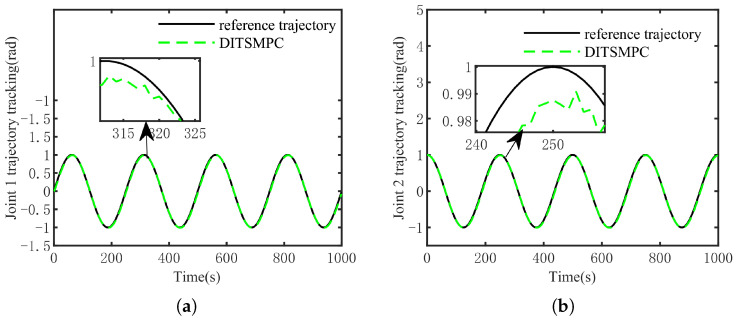
The difference in the control effect under different parameters (tracking). (**a**) Joint 1 tracking, (**b**) Joint 2 tracking.

**Figure 14:**
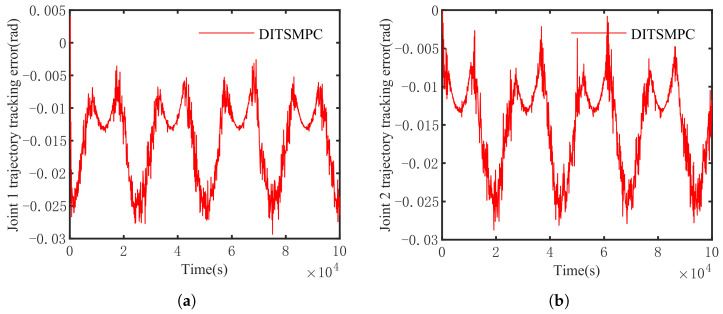
The difference in the control effect under different parameters (tracking error). (**a**) Joint 1, (**b**) Joint 2.

The simulation parameters are detailed in Table 1. The simulation parameters were obtained by a trial and error method. The following lists several important parameters on the controller performance influence diagram, as shown in Figure 13 and Figure 14, respectively. When $\lambda_{1}=1.5$, $\lambda_{2}=0.9$ and α=7/9, the remaining parameters are as shown in Table 1, and the DITSMPC performance changes.

## 6. Conclusions

As the demand for industrial robots in complex environments continues to grow, improving the posture tracking accuracy of robotic systems under unknown disturbances has become a key research topic in the field of control. This paper addresses the posture tracking control problem of robotic arms under unknown disturbances by proposing a novel DITSMPC scheme. The scheme integrates the Lagrangian dynamic analysis method with sliding mode control techniques, effectively addressing unknown disturbances and discretization errors through adaptive switching terms and delay estimation. Furthermore, it enhances the system’s stability and tracking accuracy by incorporating predictive control.

Our experimental results demonstrate that the proposed DITSMPC scheme exhibits smooth control torque variations in multi-joint robotic arm control and effectively suppresses chattering phenomena that are typically observed in traditional sliding mode control.

Subsequent research based on this paper will address the following: (1) Considering the multi-degree-of-freedom robotic arm model as the research object [27], the effectiveness of this control scheme in complex environments will be investigated. (2) Considering the incorporation of state observers for disturbance estimation [28], the current one-step delay estimation method in the algorithm may not fully estimate disturbances. Therefore, the use of state observers or disturbance observers for disturbance estimation will be explored to further improve the algorithm’s disturbance rejection capability.

## Figures and Tables

**Table 1:** Simulation parameters.

Parameter	Parameter Value
$\lambda_{1}$	0.975
$\lambda_{2}$	1.247
α	15/21
ς	98
β	0.04
$T_{s}$	0.01 s
Y	4500
N	10

**Table 2:** Performance indexes.

Control Method	MSE	IAFV
ADITSMC	$3.24\times 10^{-6}$	$6.97\times 10^{3}$
DITSMC	$6.75\times 10^{-5}$	$1.85\times 10^{4}$
DITSMPC	$2.08\times 10^{-6}$	$5.22\times 10^{3}$

## Data Availability

The data presented in this study are available in the article.
